# Neighborhood socioeconomic position, living arrangements, and cardiometabolic disease among older Puerto Ricans: An examination using PREHCO 2002–2007

**DOI:** 10.1371/journal.pone.0289170

**Published:** 2023-08-01

**Authors:** Nekehia T. Quashie, Catherine García, Gabriella Meltzer, Flavia C. D. Andrade, Amílcar Matos-Moreno

**Affiliations:** 1 Department of Health Studies, University of Rhode Island, Kingston, RI, United States of America; 2 Department of Human Development and Family Science, Aging Studies Institute, Center for Aging and Policy Studies, Lerner Center for Public Health Promotion and Population Health, Syracuse University, Syracuse, NY, United States of America; 3 Departments of Epidemiology and Environmental Health Sciences, Columbia University Mailman School of Public Health, New York, NY, United States of America; 4 School of Social Work, University of Illinois, Urbana-Champaign, Urbana, IL, United States of America; 5 Population Research Institute, The Pennsylvania State University, State College, PA, United States of America; 6 Clinical Psychology Department, Carlos Albizu University, San Juan, Puerto Rico; Universidad Cientifica del Sur, PERU

## Abstract

Cardiometabolic diseases are among the leading causes of mortality worldwide and are increasingly prevalent in rapidly aging populations. Neighborhood socioeconomic position (SEP) and living arrangements are increasingly recognized as important determinants of cardiometabolic health but have not been examined within Puerto Rico. This study examined the association between neighborhood SEP, living arrangements, and incidence of cardiometabolic conditions among island-dwelling older Puerto Ricans, using longitudinal data from the Puerto Rican Elderly Health Conditions Project (Waves I 2002/03 and II 2006/07) linked with 2000 Census data for neighborhood-level conditions. Our sample consists of non-institutionalized adults aged 60 and older who remained in the same residence over both waves of data collection (N = 2,769). We used multilevel multinomial logistic regression models to examine the relationship between neighborhood SEP and the prevalence and incidence of cardiometabolic disease. Findings show that residence in a socioeconomically advantaged neighborhood was positively associated with reporting having one cardiometabolic condition at baseline, but not associated with the incidence of cardiometabolic conditions at follow-up. Living without a partner was negatively associated with reporting having cardiometabolic conditions compared to living with a partner. Similar results were found for the incidence of cardiometabolic conditions. Living arrangements significantly modified the relationship between neighborhood SEP and cardiometabolic conditions. Compared to living with a partner, living alone in a socioeconomically advantaged neighborhood was associated with a reduced risk of reporting having one condition. Living with children in a socioeconomically advantaged neighborhood was associated with a reduced risk of developing one cardiometabolic condition than living with a partner. Living arrangements are more salient to cardiometabolic health than neighborhood SEP. Social programs and services focused on household composition and familial support are needed to identify older Puerto Ricans potentially at risk of underdiagnosed chronic conditions, especially as ongoing economic, demographic, environmental, and healthcare crises potentially exacerbate social inequalities.

## Introduction

Cardiometabolic diseases, including cardiovascular diseases (e.g., heart disease and stroke) and diabetes, are leading causes of mortality and disability worldwide [1]. The cardiometabolic disease burden is increasingly prevalent, and a major public health challenge, within Latin American and Caribbean (LAC) countries whose populations are rapidly aging [2]. Puerto Rico, an unincorporated U.S. territory with the most advanced aging within the LAC region [3], has a high prevalence of cardiometabolic diseases, with hypertension and diabetes being more prevalent among Puerto Rican older adults than older adults on the U.S. mainland [4]. Emerging research among older adults in Puerto Rico suggests that socioeconomic conditions are important determinants of later-life cardiometabolic risks [5, 6]. Despite growing evidence that social environments, including neighborhood and household contexts, are also critical social determinants of later-life cardiometabolic health [7–9], these factors have not been examined among older adults in Puerto Rico. This study, therefore, aimed to investigate the associations between the neighborhood socioeconomic environment, living arrangements, and cardiometabolic risks among community-dwelling older adults in Puerto Rico.

## Background

The neighborhood socioeconomic (SES) environment is hypothesized to differentially influence cardiometabolic health through multiple pathways, reflecting inequalities in physical (built), material, and social resources [10]. Relative to higher SES neighborhoods, poorer neighborhoods typically have fewer spaces to create opportunities for social interaction and tend to be less socially cohesive [11]. Residents of lower SES neighborhoods also have lower physical activity, poorer diet quality, and limited access to health and social services [12]. Additionally, poorer neighborhoods have higher exposure to social and environmental stressors, including crime, poor air quality, and crowding, which accumulate over time and contribute to higher allostatic load that is also associated with higher risks of chronic conditions and cardiovascular disease mortality [13–15].

A vast body of research in the U.S. suggests that living in neighborhoods of lower compared to higher SES is associated with higher risks of several cardiometabolic (and other chronic) conditions and related mortality, including cardiovascular diseases, cancer, hypertension, diabetes, and metabolic syndrome [8, 16–20]. For instance, being born in a low SES neighborhood is associated with higher blood pressure and residing in disadvantaged neighborhoods during adulthood is linked to a higher body mass index (BMI) [21]. Living in neighborhoods with higher deprivation levels is also associated with higher weight gain [19]. Neighborhood characteristics, such as better access to healthy foods, are associated with lower premature cardiovascular mortality. However, these associations do not persist once accounting for neighborhood poverty and racial composition [22], suggesting that the health benefits of higher-quality neighborhood conditions do not necessarily reduce socioeconomic disparities in some contexts.

A few empirical studies also report inconclusive (positive and negative) and null associations between neighborhood SES, health outcomes, and healthcare access. Using longitudinal data from California, Stoddard and colleagues found that adults with diabetes residing in the least-deprived neighborhoods had the lowest changes in BMI, but those living in areas with higher deprivation experienced both weight loss and weight gain at higher rates [23]. In a sample of adults in Philadelphia, Hussein and colleagues [24] found no statistically significant differences in overall healthcare access across neighborhoods. Nevertheless, the same study showed neighborhood disparities in types of healthcare access, such that residents in lower SES neighborhoods were less reliant on physician visits but more reliant on community health centers and outpatient clinics [24]. Furthermore, a longitudinal study of Puerto Rican adults in Boston found that neighborhood socioeconomic status was unrelated to changes in allostatic load [25].

Despite mixed evidence, the literature generally suggests that residents of lower compared to higher SES neighborhoods tend to have higher risks for cardiometabolic conditions due to poor structural conditions, chronic stressors, and fewer social resources to buffer stress. However, results are influenced by neighborhood characteristics (racial composition and poverty), geographic setting (with some being regional in scope, thereby limiting generalizability), and study design, with fewer longitudinal relative to cross-sectional designs. Therefore, there is a need for longitudinal studies to explore how neighborhood SES influences cardiometabolic risk factors and health.

Within neighborhoods, older adults may differ in their responses to perceived or actual stressors in their surroundings. One potential explanation is that their living arrangement, a primary daily social environment that can be a source of social support or strain, shapes older adults’ health [26]. For example, coresidence (or living) with family can positively affect health by fostering social integration and providing support, such as pooling and sharing economic resources, assistance with personal health and care needs, and companionship. These factors can help alleviate stress and improve overall well-being [27]. Family members may also monitor healthy lifestyle habits (social control), which can lead to improved health [28]. Shared households, however, can also increase opportunities for interpersonal conflicts, or household members may be unsupportive, which can negatively impact health [29].

Empirical evidence on the relationship between living arrangements and later life health is inconclusive. A volume of research suggests that living with a partner in old age is beneficial for health due to the presence of both social support and social control mechanisms. Partnership provides access to a wider array of social support (including financial, instrumental, and social resources) and a source to monitor one’s health status and encourage health seeking [30]. Furthermore, as partners share similar environments, there is strong concordance in health behaviors that can enhance health through adopting healthier lifestyle habits [31, 32] or diminish health, including presenting elevated risk factors (e.g., diet, exercise, BMI, smoking, cholesterol, and glucose levels) for cardiovascular disease [31, 33]. Thus, living with a partner can positively and negatively impact health.

Nevertheless, living alone (or without a partner) is typically associated with higher health risks than living with a partner, including unhealthy lifestyle behaviors [34], lower adherence to treatment for chronic conditions such as hypertension [35], and risks of chronic conditions including cardiovascular diseases [7, 36, 37]. Yet, other studies suggest that living alone (compared to living with others) presents health advantages for community-dwelling older adults, particularly among the oldest old [38–40].

Empirical evidence on the health benefits of living with others, such as children and/or other non (kin), suggests that multigenerational living arrangements are associated with lower risks of poor health for older adults, especially in societies with limited formal support systems [41, 42]. These findings partially reflect sociocultural dimensions such as stronger social norms of family support and the reliance on informal support that boosts and maintains health. Coresidence with children can also promote more frequent healthcare use [43] and financial assistance with medical expenses [44]. Therefore, older adults living with a partner or others may be less dependent on their neighborhoods for social support than those living alone.

Neighborhood SES, as an indicator or proxy of the quality of available amenities and resources, including social conditions, may be especially salient for the health of older adults living alone. There is a scarcity of research exploring whether older adults’ living arrangements modify the association between neighborhood conditions and health. The existing studies are predominantly centered on mental health or quality of life. Studies across diverse populations, including the U.S., Ghana, and Hong Kong, indicate that older adults living alone in more disadvantaged neighborhoods, as measured by social cohesion and the quality of the built environment, are more likely to experience poor mental health (e.g., depression, psychological distress) relative to those living with others [45–47]. However, one cross-sectional study that examined physical health outcomes found that neighborhood context (social cohesion) did not modify the association between living arrangements and chronic conditions among older Chinese American adults [48]. Although our current study cannot examine qualitative aspects of neighborhoods, such as social cohesion or the physical environment, neighborhoods’ social and physical features generally align with the neighborhood’s SES. Thus, the existing studies provide initial evidence that both living alone and with others in more (versus less) advantaged neighborhoods potentially provides better opportunity structures to enhance some dimensions of later-life health.

Understanding the importance of neighborhood and household contexts for the cardiometabolic health risks of island-dwelling Puerto Ricans is of urgent public health relevance. Puerto Rico has endured social and economic challenges from the late 20^th^ century to the beginning of the 21^st^ century, which potentially (in)directly shape current inequalities in social environments and the health of older Puerto Ricans on the island. These challenges include the mass migration of Puerto Ricans to the U.S. in the 1960s and 1970s, an increased crime rate and violence in the 1990s, a decrease in the total population in the 2000s, the furlough of public employees, and a fiscal crisis in 2006 that led to a mass outmigration of working-age adults to name a few. Macro-contextual challenges, compounded by inadequate policies and a lack of access to resources, have aggravated the health and well-being of people and communities, particularly older adults [49–52]. The healthcare system has also been impacted, evidenced by the unequal distribution of healthcare services that favor socioeconomically advantaged areas, increasing migration of healthcare professionals, and entrenching inequality in health services covered by Medicaid and Medicare [16, 52].

In addition to socioeconomic conditions, living arrangements and household composition may further influence older Puerto Rican adults’ health. Declining fertility and continued outmigration of younger cohorts to the U.S. mainland limits the availability of adult children, the traditional source of support, presenting challenges for informal care to sustain older Puerto Ricans’ health [53]. Although living alone or with a spouse is increasingly common in Puerto Rico [54], there is a lack of research on health consequences for older adults. Prior research has shown that older adults living with a partner were less likely than those living alone to receive support, particularly health-related, from children [55]. While older adults living with their partners may be able to access support when health (or other) needs arise, those living alone or without a partner may be more vulnerable to health risks. Yet, health risks linked to living arrangements may vary across neighborhood SES conditions, given residential segregation and the unequal distribution of healthcare resources across Puerto Rico. Older adults living alone or without a partner in more advantaged neighborhoods may experience better health overall through better access to resources relative to living in a more deprived neighborhood.

Therefore, drawing upon longitudinal data from the Puerto Rican Elderly Health Conditions (PREHCO) project, we extend the existing research on neighborhood SES, living arrangements, and health by examining the effects (independent and interactive) on cardiometabolic health among older adults in Puerto Rico. We evaluate three hypotheses. First, older adults in lower versus higher SES neighborhoods will have a higher risk of reporting prevalent and incident cardiometabolic diseases (H_1_). Second, older adults living alone will have the highest risk of prevalent and incident cardiometabolic conditions relative to older adults living with a partner (H_2_). Third, living arrangements will moderate the association between neighborhood socioeconomic position and cardiometabolic health (H_3_).

## Materials and methods

### Data and sample selection

We used data from the Puerto Rican Elderly Health Conditions (PREHCO) Project, a longitudinal study of community-dwelling adults 60 years and older who resided in the main island of Puerto Rico at the time of the study [56]. PREHCO baseline data (Wave 1) were collected via 4,291 face-to-face interviews between 2002–2003, and a follow-up (Wave 2) of 3,891 face-to-face interviews was conducted between 2006–2007. Both waves yielded a response rate of over 90%, and prior studies provide information on the overall design, sampling procedures, and survey instruments of PREHCO [56, 57]. The present study participants represent those who stayed in the same residence across both waves (n = 2,867) as stable residents are likely to be exposed to a consistent neighborhood SES, thereby providing the opportunity to establish a clearer longitudinal association between neighborhood SES and cardiometabolic incidence. Results of chi-square tests indicate that relative to those who maintained stable residence, participants who moved between the waves were more likely to be living without a partner (alone (31% vs. 23%); with children (21% vs 15%); or with others (13% vs 8%), *p* < 0.002)), and slightly older (80 years and above (*p* < 0.02)). There were no statistically significant group differences by neighborhood SEP (socioeconomic position), gender, educational attainment, and health insurance coverage. We excluded respondents with missing data on the variables included in our analysis (n = 98). Thus, our analytic sample includes 2,769 older adults.

Data from the 2000 U.S. Decennial Census were downloaded from the Puerto Rico Contextual Data Resource (PR-CDR) [58] to create a baseline measure of the neighborhood socioeconomic environment at the block group level, consisting of 2,477 block groups. These data were then combined with the individual-level PREHCO data using Federal Information Processing Standard (FIPS) codes to link the files. All 2,769 respondents from our analytic sample were matched and represented 229 census block groups.

PREHCO data were collected by the University of Wisconsin-Madison and the University of Puerto Rico. The PR-CDR was developed at Syracuse University in collaboration with the University of Puerto Rico and the University of Alabama at Birmingham. Participation in the study was voluntary, and written and oral informed consent was obtained. These data are publicly available in anonymized forms [56]. All methods used in our study rely on these anonymized secondary data. This study was deemed exempt and met the ethical standards set by the institutional review board of Syracuse University (IRB #21–217).

### Measures

#### Cardiometabolic conditions

In Wave 1, respondents self-reported the presence of doctor-diagnosed hypertension, diabetes, and cardiovascular diseases (heart disease including angina, coronary heart disease, congestive heart disease, heart attack, and stroke). At Wave 2, respondents only reported newly diagnosed conditions since Wave 1. Following previous studies [17], we utilized information on self-reported conditions at both waves to create a categorical variable that assessed the prevalence of existing conditions at both waves and the incidence of health conditions at Wave 2. The variable represented respondents within five mutually exclusive categories: 1) no health conditions at baseline and follow-up, 2) one existing condition at baseline and follow-up, 3) two or more existing conditions at baseline and follow-up, 4) one incident condition at follow-up, and 5) two or more incident conditions at follow-up.

#### Neighborhood socioeconomic environment

To define the neighborhood socioeconomic environment, we employed the socioeconomic position (SEP) index, consisting of eight census-based variables at the block group level using the methods described by Torres-Cintrón and colleagues to assess cancer incidence and mortality in Puerto Rico [16] (see Table 1). The continuous SEP index was then converted into a variable consisting of quintile categories ranging from 1 (least socioeconomically deprived; High SEP) to 5 (most socioeconomically deprived; Low SEP).

**Table 1:** Principal Component (PC) score coefficients to define the Socioeconomic Position (SEP) index derived at the block group level, Puerto Rico, 2000.

Name of Variable	PC Score Coefficient
Unemployment rate	0.3283
Median household income^a^	0.3995
% Living below poverty	0.3807
% <12 years of education	0.3806
% Occupied housing units without a car	0.3059
% Employed in management, professional, & related occupations^a^	0.3332
% Occupied housing units w/o a telephone	0.3604
% Population fluent in both English and Spanish^a^	0.3291

^a^Values were reverse coded before the z score was computed so that a higher score corresponded to a lower socioeconomic position index score.

#### Living arrangements

We view living arrangements, specifically the household composition, as an indication of older adults’ source of social relationships that may enhance or impair their health. We constructed respondents’ baseline living arrangements based on information provided on the relationship to their household members and categorized them as 1) living with spouse/partner only, 2) living alone, 3) living with children only, 4) living with others including other family (e.g., parents, siblings) and non-family members. As documented in prior research [55], living arrangements and marital status are inseparable within Puerto Rico. In the current study, three respondents were also partnered among those living with children only (0.55%). Thus, most older adults living alone, with children, or with others were unpartnered.

#### Covariates

Baseline sociodemographic characteristics and socioeconomic resources were selected as confounding factors. Sociodemographic characteristics included reported age measured in five-year increments: 60–64 years (reference), 65–69, 70–74, 75–79, 80–84, and 85+; and gender (women/men). Socioeconomic indicators included educational attainment (less than high school (reference), high school, college and beyond); and health insurance coverage categorized as Medicaid (reference), Medicare Part A or B, private or employment-based (e.g., private plan, teacher, police, etc.) or uninsured.

#### Statistical analysis

First, we present a description of the analytic sample showing the distribution of respondents’ characteristics at baseline. We also give estimates for the prevalence and incidence of chronic health conditions. Multilevel multinomial logistic regression was performed via a generalized structural equation modeling approach (with a logit link function) to document the association between neighborhood SEP, living arrangements, and cardiometabolic conditions with no health conditions as the reference category. The coefficients are presented as relative probability ratios (or relative risk ratios) with 95% confidence intervals. We additionally include a graphical presentation of the predicted probabilities of the final model for ease of interpretation. Our base model included neighborhood SEP, age, and sex (Model 1). Next, we included living arrangements (Model 2) before adding our socioeconomic controls (Model 3). Finally, we examined the interaction between neighborhood SEP and living arrangements (Model 4). We accounted for the complex survey design of PREHCO by adjusting for sample weights and stratification variables. Analyses were conducted using Stata version 17.0/MP [59].

## Results and discussion

### Distribution of neighborhood SEP in Puerto Rico

**Fig 1:**
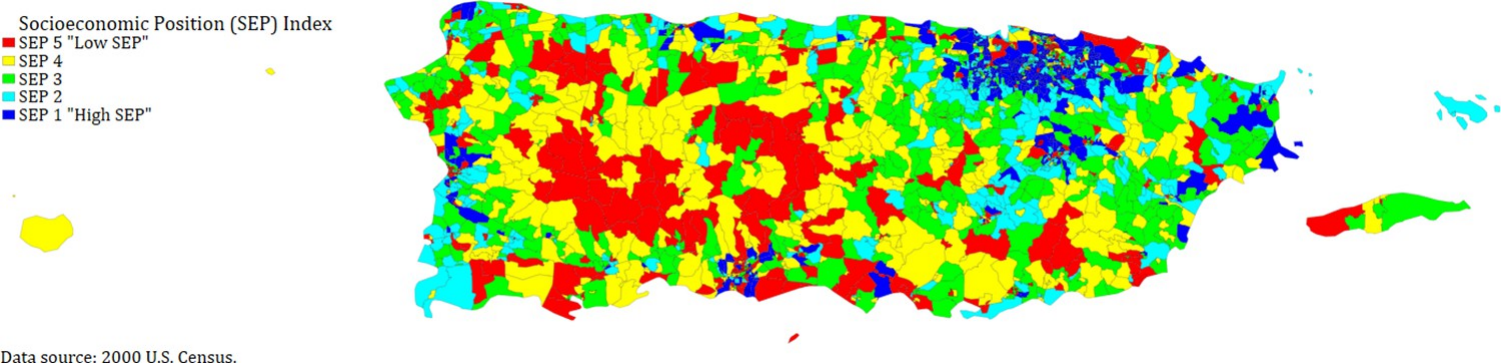
Block groups by Socioeconomic Position (SEP) index, Puerto Rico, 2000.

Lower SEP neighborhoods can be found along the central and coastal areas of Puerto Rico (Fig 1). Higher SEP neighborhoods tend to be found in the northeast and coastal areas.

### Sample characteristics

Table 2 describes the characteristics of our analytic sample, including the distribution of baseline and follow-up cardiometabolic conditions. Overall, at baseline, most older Puerto Ricans in our sample (approximately 55%) resided in neighborhoods with high to moderate socioeconomic deprivation (i.e., neighborhood SEP 3 to 5). Regarding living arrangements, older adults primarily lived with their partners (54%) or alone (22%). About two-thirds of older adults were between 60 and 69 years, and more than half of the respondents were women (57%). Respondents generally had low levels of education, with 65% having attained less than a high school education. Yet, most older adults had health insurance coverage, with nearly half covered by government insurance (49%).

**Table 2:** Descriptive statistics for the Puerto Rican Elderly Health Conditions project (PREHCO) sample.

Variable	%^a^
**Baseline Neighborhood SEP**	
1 “High SEP”	21.6
2	23.1
3	22.0
4	17.0
5 “Low SEP”	16.2
**Baseline Living Arrangement**	
With spouse/partner	54.5
Alone	22.2
With children	15.0
With others (family/non-family)	8.3
**Age**	
60–64	31.3
65–69	25.6
70–74	19.5
75–79	12.9
80–84	6.5
85+	4.4
**Gender**	
Women	57.1
Men	43.0
**Baseline Education**	
Less than high school	65.4
High school	21.2
College and beyond	13.4
**Baseline Health Insurance**	
Government plan	49.0
Medicare Part A or B	33.0
Private or employment-based	15.3
Uninsured	2.7
**Baseline Cardiometabolic Conditions**	
Hypertension	57.7
Diabetes	26.1
Heart disease	16.6
Heart attack	9.2
Stroke	5.0
**Longitudinal Cardiometabolic Conditions**	
Zero conditions at baseline & follow-up	19.8
1 existing condition at baseline & follow-up	25.7
2 existing conditions at baseline & follow-up	23.9
1 incident condition at follow-up	23.9
2+ incident conditions at follow-up	6.7
Unweighted N	2,769

^a^Weighted percentages

Note: SEP = socioeconomic position

When examining the prevalence of each cardiometabolic condition at baseline, over half of the respondents reported having hypertension (58%), and a few reported having experienced a stroke (5%). Looking at the prevalence and incidence of cardiometabolic conditions, 20% of older adults reported no conditions across both waves. Most older adults, however, reported one (26%) or two (24%) conditions at baseline and follow-up. Nearly one-quarter of the sample developed one new condition by wave 2 (24%), and a relatively small share of older adults developed two or more conditions at follow-up (7%).

### Associations between neighborhood SEP, living arrangements, and cardiometabolic conditions

Tables 3–6 show the estimated results of the multilevel multinomial logistic regression models presented as relative risk ratios (RRRs) with 95% confidence intervals (CIs). RRRs between zero and one indicate a reduction in the risk of reporting current and incident cardiometabolic conditions. RRRs greater than one indicates an increase in the risk of reporting current and incident cardiometabolic conditions. Model 1 shows that living in more socioeconomically deprived neighborhoods was associated with an increased risk of reporting an existing cardiometabolic condition–exactly one (SEP_2_, RRR = 1.46, 95% CI [1.02, 2.08]) and two or more (SEP_3_, RRR = 1.44, 95% CI [1.00, 2.08])–and developing one incident condition (SEP_2_, RRR = 1.49, 95% CI [1.04, 2.13]; SEP_4_, RRR = 1.61, 95% CI [1.10, 2.35]; SEP_5_, RRR = 1.45, 95% CI [1.02, 2.05]) relative to those who reported having no health conditions at baseline and follow-up. This association persisted even after adjusting for living arrangements (Model 2). After adjusting for educational attainment and type of health insurance (Model 3), the association between neighborhood SEP and cardiometabolic conditions was attenuated; neighborhood SEP 2 maintained a significant association with having one existing cardiometabolic condition (RRR = 1.49, 95% CI [1.04, 2.13]). Neighborhood SEP was unrelated to the incidence of cardiometabolic conditions. Overall, the data do not support our hypothesis (H_1_) that residence in socioeconomically disadvantaged neighborhoods is associated with an increased risk of reporting current or incident cardiometabolic conditions.

**Table 3:** Estimates of the multilevel multinomial logistic regression models for one existing condition versus none^†^.

	M1		M2		M3		M4	
	RRR	95% CI	RRR	95% CI	RRR	95% CI	RRR	95% CI
**Neighborhood SEP (ref = 1 "High SEP")**								
2	1.46 *	[1.02,2.08]	1.48 *	[1.03,2.12]	1.49*	[1.04,2.13]	2.20**	[1.25,3.88]
3	1.11	[0.77,1.61]	1.14	[0.79,1.65]	1.14	[0.78,1.66]	1.59	[0.89,2.86]
4	1.29	[0.88,1.89]	1.31	[0.89,1.93]	1.26	[0.85,1.88]	1.46	[0.82,2.60]
5	1.03	[0.72,1.47]	1.09	[0.76,1.56]	1.05	[0.72,1.53]	1.20	[0.67,2.15]
**Living Arrangement (ref = with spouse/partner)**								
Alone			0.63**	[0.46,0.85]	0.63**	[0.47,0.86]	0.97	[0.53,1.78]
With children			0.52***	[0.37,0.74]	0.52***	[0.36,0.74]	0.80	[0.39,1.62]
With others			0.66*	[0.44,1.00]	0.66*	[0.44,1.00]	0.53	[0.21,1.33]
**Neighborhood SEP x Living Arrangement**								
** Neighborhood SEP 2**								
** **Neighborhood SEP 2 x Living alone							0.40*	[0.17,0.94]
** **Neighborhood SEP 2 x Living with children							0.57	[0.22,1.49]
** **Neighborhood SEP 2 x Living with others							0.93	[0.27,3.17]
** Neighborhood SEP 3**								
** **Neighborhood SEP 3 x Living alone							0.52	[0.22,1.22]
** **Neighborhood SEP 3 x Living with children							0.66	[0.24,1.84]
** **Neighborhood SEP 3 x Living with others							0.61	[0.17,2.23]
** Neighborhood SEP 4**								
** **Neighborhood SEP 4 x Living alone							0.61	[0.24,1.55]
** **Neighborhood SEP 4 x Living with children							0.66	[0.24,1.80]
** **Neighborhood SEP 4 x Living with others							2.59	[0.61,11.00]
** Neighborhood SEP 5**								
** **Neighborhood SEP 5 x Living alone							0.82	[0.35,1.92]
** **Neighborhood SEP 5 x Living with children							0.47	[0.18,1.23]
** **Neighborhood SEP 5 x Living with others							2.02	[0.59,6.93]

Note: SEP = socioeconomic position; RRR = relative risk ratio; CI = confidence interval

* p < .05

** p < .01

*** p < .001

Controls: age, gender, educational attainment, and type of health insurance.

^†^Classification accuracy = 0.7071145.

**Table 4:** Estimates of the multilevel multinomial logistic regression models for two existing conditions versus none.

	M1		M2		M3		M4	
	RRR	95% CI	RRR	95% CI	RRR	95% CI	RRR	95% CI
**Neighborhood SEP (ref = 1 "High SEP")**								
2	1.16	[0.80,1.69]	1.18	[0.81,1.73]	1.13	[0.78,1.64]	1.33	[0.74,2.37]
3	1.44**	[1.00,2.08]	1.50*	[1.04,2.18]	1.37	[0.94,1.99]	1.75	[0.99,3.09]
4	1.31	[0.89,1.94]	1.33	[0.89,1.98]	1.13	[0.75,1.69]	1.19	[0.67,2.12]
5	1.23	[0.86,1.75]	1.32	[0.92,1.90]	1.14	[0.79,1.67]	1.04	[0.58,1.85]
**Living Arrangement (ref = with spouse/partner)**								
Alone			0.56***	[0.41,0.76]	0.55***	[0.40,0.75]	0.60	[0.32,1.12]
With children			0.50***	[0.35,0.71]	0.47***	[0.33,0.67]	0.58	[0.28,1.20]
With others			0.51**	[0.33,0.78]	0.50**	[0.32,0.76]	0.48	[0.19,1.20]
**Neighborhood SEP x Living Arrangement**								
** Neighborhood SEP 2**								
** **Neighborhood SEP 2 x Living alone							0.82	[0.33,1.99]
** **Neighborhood SEP 2 x Living with children							0.78	[0.28,2.13]
** **Neighborhood SEP 2 x Living with others							0.63	[0.17,2.44]
** Neighborhood SEP 3**								
** **Neighborhood SEP 3 x Living alone							0.54	[0.23,1.30]
** **Neighborhood SEP 3 x Living with children							0.71	[0.25,1.97]
** **Neighborhood SEP 3 x Living with others							1.02	[0.30,3.44]
** Neighborhood SEP 4**								
** **Neighborhood SEP 4 x Living alone							0.95	[0.37,2.44]
** **Neighborhood SEP 4 x Living with children							0.81	[0.29,2.23]
** **Neighborhood SEP 4 x Living with others							1.06	[0.21,5.27]
** Neighborhood SEP 5**								
** **Neighborhood SEP 5 x Living alone							1.39	[0.59,3.28]
** **Neighborhood SEP 5 x Living with children							0.80	[0.31,2.06]
** **Neighborhood SEP 5 x Living with others							1.44	[0.41,5.09]

Note: SEP = socioeconomic position; RRR = relative risk ratio; CI = confidence interval

* p < .05

** p < .01

*** p < .001

Controls: age, gender, educational attainment, and type of health insurance.

^†^Classification accuracy = 0.7071145.

**Table 5:** Estimates of the multilevel multinomial logistic regression models for one incident condition versus none.

	M1		M2		M3		M4	
	RRR	95% CI	RRR	95% CI	RRR	95% CI	RRR	95% CI
**Neighborhood SEP (ref = 1 "High SEP")**								
2	1.49*	[1.04,2.13]	1.51*	[1.05,2.16]	1.35	[0.93,1.96]	1.97*	[1.10,3.50]
3	1.23	[0.85,1.77]	1.27	[0.87,1.83]	1.06	[0.72,1.57]	1.29	[0.70,2.36]
4	1.61*	[1.10,2.35]	1.62*	[1.11,2.36]	1.28	[0.85,1.93]	1.30	[0.72,2.35]
5	1.45*	[1.02,2.05]	1.53*	[1.08,2.17]	1.22	[0.84,1.79]	1.46	[0.82,2.60]
**Living Arrangement (ref = with spouse/partner)**								
Alone			0.79	[0.59,1.07]	0.76	[0.56,1.03]	0.94	[0.49,1.78]
With children			0.65*	[0.46,0.93]	0.60**	[0.42,0.86]	1.09	[0.53,2.23]
With others			0.49**	[0.31,0.76]	0.48**	[0.30,0.75]	0.34	[0.11,1.03]
**Neighborhood SEP x Living Arrangement**								
** Neighborhood SEP 2**								
** **Neighborhood SEP 2 x Living alone							0.66	[0.27,1.58]
** **Neighborhood SEP 2 x Living with children							0.35*	[0.13,0.96]
** **Neighborhood SEP 2 x Living with others							0.52	[0.11,2.51]
** Neighborhood SEP 3**								
** **Neighborhood SEP 3 x Living alone							0.73	[0.30,1.78]
** **Neighborhood SEP 3 x Living with children							0.55	[0.19,1.58]
** **Neighborhood SEP 3 x Living with others							1.51	[0.37,6.27]
** Neighborhood SEP 4**								
** **Neighborhood SEP 4 x Living alone							1.11	[0.44,2.80]
** **Neighborhood SEP 4 x Living with children							0.69	[0.26,1.86]
** **Neighborhood SEP 4 x Living with others							1.36	[0.23,8.00]
** Neighborhood SEP 5**								
** **Neighborhood SEP 5 x Living alone							0.73	[0.31,1.74]
** **Neighborhood SEP 5 x Living with children							0.44	[0.17,1.11]
** **Neighborhood SEP 5 x Living with others							2.58	[0.65,10.24]

Note: SEP = socioeconomic position; RRR = relative risk ratio; CI = confidence interval

* p < .05

** p < .01

*** p < .001

Controls: age, gender, educational attainment, and type of health insurance.

^†^Classification accuracy = 0.7071145.

**Table 6:** Estimates of the multilevel multinomial logistic regression models for two or more incident conditions versus none.

	M1		M2		M3		M4	
	RRR	95% CI	RRR	95% CI	RRR	95% CI	RRR	95% CI
**Neighborhood SEP (ref = 1 "High SEP")**								
2	1.28	[0.71,2.31]	1.31	[0.73,2.36]	1.1	[0.60,1.99]	1.72	[0.71,4.18]
3	1.67	[0.95,2.93]	1.74	[0.99,3.06]	1.33	[0.74,2.38]	2.14	[0.90,5.09]
4	1.67	[0.93,3.02]	1.68	[0.93,3.02]	1.20	[0.65,2.23]	1.66	[0.69,3.95]
5	1.63	[0.94,2.81]	1.72	[1.00,2.99]	1.23	[0.69,2.21]	1.43	[0.59,3.47]
**Living Arrangement (ref = with spouse/partner)**								
Alone			0.57*	[0.36,0.91]	0.53**	[0.33,0.84]	0.65	[0.21,2.08]
With children			0.70	[0.41,1.19]	0.62	[0.36,1.05]	1.46	[0.50,4.31]
With others			0.58	[0.30,1.12]	0.55	[0.28,1.07]	1.04	[0.25,4.31]
**Neighborhood SEP x Living Arrangement**								
** Neighborhood SEP 2**								
** **Neighborhood SEP 2 x Living alone							0.87	[0.20,3.87]
** **Neighborhood SEP 2 x Living with children							0.19	[0.04,1.06]
** **Neighborhood SEP 2 x Living with others							0.35	[0.04,2.97]
** Neighborhood SEP 3**								
** **Neighborhood SEP 3 x Living alone							0.47	[0.11,2.06]
** **Neighborhood SEP 3 x Living with children							0.43	[0.10,1.89]
** **Neighborhood SEP 3 x Living with others							0.41	[0.06,2.71]
** Neighborhood SEP 4**								
** **Neighborhood SEP 4 x Living alone							0.79	[0.17,3.74]
** **Neighborhood SEP 4 x Living with children							0.43	[0.10,1.87]
** **Neighborhood SEP 4 x Living with others							0.00	[0.00,0.00]
** Neighborhood SEP 5**								
** **Neighborhood SEP 5 x Living alone							1.16	[0.28,4.84]
** **Neighborhood SEP 5 x Living with children							0.40	[0.10,1.61]
** **Neighborhood SEP 5 x Living with others							0.98	[0.15,6.20]

Note: SEP = socioeconomic position; RRR = relative risk ratio; CI = confidence interval

* p < .05

** p < .01

*** p < .001

Controls: age, gender, educational attainment, and type of health insurance.

^†^Classification accuracy = 0.7071145.

Regarding living arrangements, Models 2 and 3 showed that older adults who were not living with a partner (i.e., alone, with children, and with others) was associated with a 34%-50% reduction in reporting one (alone, RRR = 0.63, 95% CI [0.47, 0.86]; with children, RRR = 0.52, 95% CI [0.36, 0.74]; with others, RRR = 0.66, 95% CI [0.44, 1.00]) or two existing cardiometabolic conditions (alone, RRR = 0.55, 95% CI [0.40, 0.75]; with children, RRR = 0.47, 95% CI [0.33, 0.67]; with others, RRR = 0.50, 95% CI [0.32, 0.76]). Living with children (RRR = 0.60, 95% CI [0.42, 0.86]) and with others (RRR = 0.48, 95% CI [0.30, 0.75]) was associated with a 35%-51% reduction in reporting an incident condition as opposed to living with a partner. Living alone was associated with a 47% reduction in reporting two or more incident conditions over the follow-up period relative to living with a partner (RRR = 0.53, 95% CI [0.33, 0.84]). Thus, the findings do not support our hypothesis (H_2_) that older adults living alone have an increased risk of reporting current and incident cardiometabolic conditions.

**Fig 2:**
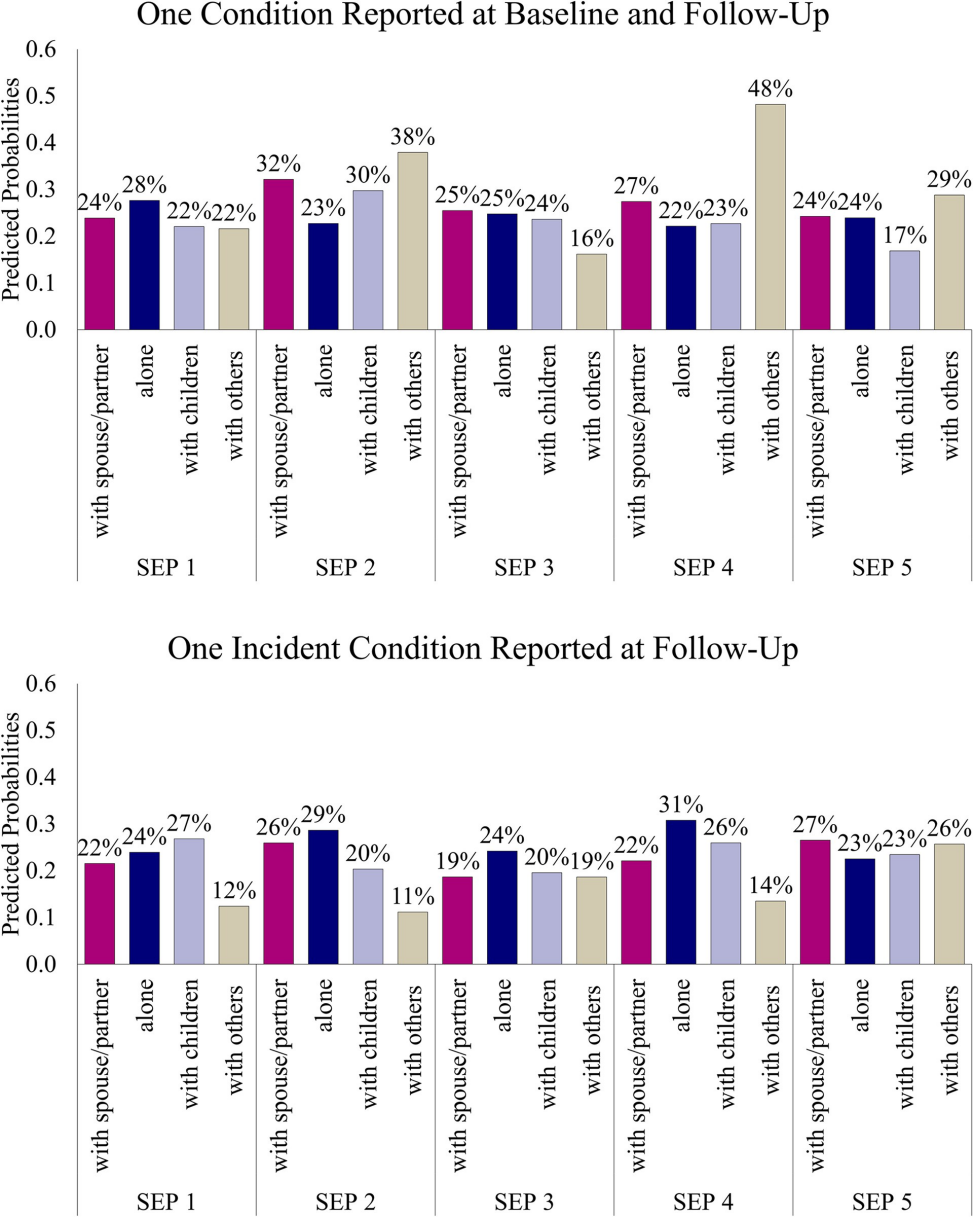
The predicted probabilities of reporting one existing and the incidence of one cardiometabolic condition according to living arrangement across each neighborhood socioeconomic position.

Next, we examined whether older adults’ living arrangements moderated the association between neighborhood SEP and health. Our results showed that living arrangements significantly moderated the association between neighborhood SEP and reporting one existing and one incident condition but with variation by household composition. To facilitate the interpretation of the moderation, Fig 2 shows the predicted probabilities of reporting one existing condition and the incidence of reporting one cardiometabolic condition according to living arrangement across each neighborhood SEP. Compared to those living with a partner, older adults living alone in neighborhood SEP 2 (a fairly advantaged neighborhood) were less likely to have one existing condition. Likewise, older adults living with children in neighborhood SEP 2 were less likely to develop one new condition.

## Discussion

The current study is the first to examine the independent and joint associations between the neighborhood socioeconomic environment, living arrangements, and cardiometabolic health using longitudinal data for a representative sample of island-dwelling Puerto Rican older adults. Overall, neighborhood socioeconomic position was weakly associated with cardiometabolic conditions. Living arrangements, however, were strongly associated with cardiometabolic conditions such that living without a partner–alone, with children, or with others–was persistently negatively associated with prevalent and incident cardiometabolic conditions. Furthermore, living arrangements presented a statistically significant moderating effect on the relationship between neighborhood socioeconomic position and cardiometabolic health. These findings were consistent after adjusting for older adults’ sociodemographic and socioeconomic characteristics.

### Neighborhood socioeconomic position and cardiometabolic conditions

Contrary to our expectations (H_1_), neighborhood disadvantage did not yield a statistically significant association with prevalent or incident cardiometabolic conditions, controlling for individuals’ socioeconomic and sociodemographic characteristics. Instead, we found that living in a relatively socioeconomically advantaged neighborhood (neighborhood SEP 2) was associated with an increased risk of reporting cardiometabolic conditions relative to living in the most advantaged neighborhood (neighborhood SEP 1). Our findings contrast with much of the existing literature showing adverse health effects of residence in socioeconomically disadvantaged neighborhoods relative to advantaged ones [8, 16, 20]. Still, some studies document null findings [24, 60], including among older Puerto Rican adults on the U.S. mainland [25]. As documented in other studies, hospitals in Puerto Rico are mostly concentrated in higher SEP neighborhoods [16]. Given that our measures of cardiometabolic health reflect self-reported doctor-diagnosed conditions, our finding potentially reflects that residents in higher neighborhood SEP areas may have greater access to health and social care services that increase their awareness and management of their conditions. Alternatively, the lack of a statistically significant association between neighborhood disadvantage and cardiometabolic health may be partially due to limited access to health care, so more individuals are undiagnosed. Additionally, there may be greater homogeneity among the more disadvantaged neighborhoods in Puerto Rico, thereby minimizing variability in health risks among older adults in lower relative to higher SEP areas. We also acknowledge that our measure of neighborhood SEP is based on aggregate measures of a limited number of sociodemographic characteristics rather than including other aspects of the neighborhood environment, such as the built environment, which may be more predictive of cardiometabolic health outcomes [61].

### Living arrangements and cardiometabolic conditions

The persistent statistically significant association between living arrangements and both prevalent and incident cardiometabolic conditions suggests that the household context is more salient than the neighborhood for the cardiometabolic health of island-dwelling older Puerto Ricans. Contrary to our hypothesis (H_2_), however, we did not find evidence that living alone presented elevated risks of prevalent and incident cardiometabolic conditions. Living without a partner appeared to be health-protective for the cardiometabolic health of island-dwelling older Puerto Rican adults. While our findings are inconsistent with several studies that suggest living alone and without a partner presents myriad health risks [36, 37], they also align with studies that suggest older adults living alone maintain better health status than those living with others [38, 39], and some argue that they are health-selected into independent living. We also found that intergenerational coresidence was associated with lower risks of prevalent and incident cardiometabolic conditions relative to living with a partner. As Puerto Rican culture values family cohesion and intergenerational support, the negative association between intergenerational coresidence and cardiometabolic conditions may partially reflect that being in a traditional living arrangement enhances older adults’ overall well-being and presents more opportunities for social support, which in turn minimizes the risk of illness [41, 42]. Nevertheless, as our measure of cardiometabolic conditions refers to self-reported doctor-diagnosed conditions, we exercise caution in interpreting our findings. Coresident children may provide various forms of support and care that substitute formal health care, including reduced doctor visits [62]. Thus, older adults in intergenerational living arrangements are potentially underdiagnosed. Likewise, older adults living alone are potentially undiagnosed or underdiagnosed, as prior research suggests that older adults living alone in Puerto Rico were less likely to receive support from children than older adults living with children [55].

Our findings also suggest that older adults in Puerto Rico who are living with a partner have higher risks of reporting poor cardiometabolic health than those living without a partner. As coresident partners are often the primary sources of social support, living with a partner may encourage and facilitate healthcare utilization and subsequent diagnosis of conditions. On the contrary, the findings may reflect health concordance among partners (including unhealthy habits) and the role of poor-quality interactions within partnerships that are associated with cardiovascular health risks [33, 63]. Our findings highlight the need for future research to examine the role of health behaviors and relationship quality among partners to help explain and disentangle the relationship between living arrangements and health risks among older Puerto Ricans.

Furthermore, living arrangements significantly modified the association between neighborhood SEP and cardiometabolic health: living alone and with children in a relatively socioeconomically advantaged neighborhood was associated with a reduced risk of reporting a current and one-incident cardiometabolic condition. This finding extends previous research that has shown the health protective effects of high-quality neighborhood environments (measured by social cohesion and physical amenities) for older adults living alone [45, 46], as well as those living with other family members, including children [48]. Therefore, residing in a socioeconomically advantaged neighborhood with potentially better quality living conditions (including access to healthier foods, health care services, and potentially higher social cohesion) supports older adults living alone who may be otherwise vulnerable within a broader sociocultural environment that emphasizes family cohesion. Similarly, intergenerational coresidence in more advantaged neighborhoods likely presents multiplicative health benefits through the immediate access to social support from children within the household (including health-related information or instrumental support to manage chronic conditions) that is bolstered by community support and services.

### Strengths and limitations

Our study has several noteworthy strengths. We examined the associations between neighborhood SEP and living arrangements on older adults’ cardiometabolic health and the modifying influence of living arrangements, contributing to the limited empirical evidence on this topic [48]. Furthermore, our study adds to a marked gap in the literature examining these relationships using a longitudinal and multilevel study design that allows us to examine individual and contextual factors linked to health and minimize potential reverse causality in the associations [25]. Finally, we utilized a representative sample of community-dwelling older adults in Puerto Rico, providing critical empirical evidence on the role of social environments in both the prevalence and incidence of cardiometabolic conditions in later life.

However, this study has limitations that should be considered when interpreting the findings. First, even though the neighborhood measure considers the socioeconomic profile, it does not factor in the built environment, such as healthcare infrastructure (e.g., hospitals, clinics), availability of healthy food options, or physical amenities (e.g., parks). Additionally, we cannot explore individuals’ perceptions of their neighborhoods’ physical and social quality, which is also linked to health behaviors and status [45, 64]. Second, the PREHCO data do not allow examination of the factors that precede individuals’ baseline living arrangements and neighborhood residence that potentially confound the relationship with their cardiometabolic health. Relatedly, despite restricting the sample to individuals who remained in the same residence between waves, thereby accounting for some aspect of residential stability, we do not have information on older adults’ complete length of residence in their neighborhoods and living arrangements. Older adults who have lived in more deprived neighborhoods or without a partner for longer periods may have adapted to their living conditions, thus reducing variability in the association between neighborhood deprivation and cardiometabolic health and minimizing the health risks of living without a partner. Third, while living arrangements were stable across the waves, as documented by prior research [55], other intricate aspects of the household environment, such as the quality of social interactions (e.g., inter- and intra- generational conflicts), housing quality and structures, and individuals’ living arrangement preference potentially mediate the relationship between living arrangements and cardiometabolic health. For instance, individuals in their preferred living arrangement may experience less social strain and have better overall health relative to those not in their desired living arrangement. Furthermore, other unmeasured individual factors (e.g., healthcare utilization, nutrition, physical activity) may bias our results.

Finally, we acknowledge that our findings pertain to the living conditions of older Puerto Ricans during the early 21^st^ century and do not reflect current linkages between neighborhood SEP, living arrangements, and cardiometabolic health. It is important to note that Puerto Rico has endured significant economic, demographic, environmental, and public health challenges since 2007, which were not accounted for in our study. These challenges include increasing fiscal constraints, widening income inequality, infrastructural damage caused by natural disasters such as Hurricanes Irma and María in 2017 and earthquakes in 2019 and 2020, large-scale emigration of younger cohorts, and a deterioration in public healthcare funding and services [65]. These factors have potentially exacerbated pre-existing inequalities in neighborhood environments, healthcare access, and social support resources, leading to systemic heterogeneity in chronic stressors that can differentially impact the cardiometabolic health of older Puerto Ricans. Although our current study cannot examine the role of these recent macro-contextual social stressors, our findings provide an important baseline for future studies to examine the role of (unequal) living conditions and living arrangements in later life health among island-dwelling Puerto Ricans. These limitations provide fruitful directions for future research, which may be explored once the third wave of PREHCO data becomes publicly available.

## Conclusions

The study contributes to a growing body of literature demonstrating the importance of neighborhood and household contexts to health. Notably, the combined influence of these social environments in later life is understudied, especially within Latin America and the Caribbean. Our results show that household contexts of older adults in Puerto Rico may have a greater influence on their cardiometabolic health than their neighborhood socioeconomic environment. This underscores the importance of further research to understand how living arrangements influence cardiometabolic health. These data may offer crucial insights for researchers and policymakers who aim to enhance health promotion programs targeting older adults in poor-quality partnerships that can trigger stress, thereby increasing the risks of cardiometabolic health or further promoting supportive partnerships that facilitate healthcare utilization for diagnosis. Similarly, public healthcare and support services should focus on assisting unpartnered older adults who live alone, with children, or with others who may not have the necessary support to facilitate healthcare visits, thus facing risks of undiagnosis or underdiagnosis. Furthermore, our findings suggest avenues for future research to examine the mechanisms that shape neighborhood socioeconomic disparities in cardiometabolic health, which may be related to differential environmental exposures or access to and utilization of healthcare.
